# The assessment of insight across cultures

**DOI:** 10.4103/0019-5545.74316

**Published:** 2010

**Authors:** K. S. Jacob

**Affiliations:** Department of Psychiatry, Christian Medical College, Vellore, India

**Keywords:** Cross-cultural assessment, culture, insight, psychosis

## Abstract

The assessment of insight is a part of the routine clinical examination for people with mental illness. Such assessment, by psychiatrists, is based on the current definitions of insight, which rely on western notions of health and illness. This paper discusses the recent findings of illness perspectives of people with a variety of physical diseases and mental disorders from India. Studies on insight in schizophrenia and bipolar disorders also examined explanatory models of illness among patients, relatives, and the general population. The findings argue for the fact that the assessment of insight should be against the local cultural standards rather than universal yardsticks. The assessment of insight should evaluate awareness, attribution, and action. People with psychosis who are able to re-label their psychotic experience, offer non-delusional explanations for changes in themselves, which correspond to beliefs about illness held by the subculture, admit to the need for restitution, and seek locally available help, can be said to possess insight. The results recommend the use of universal conventions to assess insight in people with psychosis rather than the use of uniform criteria.

## INTRODUCTION

The assessment of insight in people with schizophrenia and other psychiatric illness is an important part of the mental status examination. Studies of schizophrenia in different regions of the world have documented “lack of insight” across cultures.[1] However, the issues related to insight are complex and research argues that the traditional all-or-none approach and even the more recent recommendations, which employ universal criteria across multiple dimension, need re-evaluation.

### Defining insight

Many attempts at defining insight have been recorded in the literature. Some current definitions of insight include the following.

- “The conscious recognition of one’s own condition.”[2]
- “The degree of personal awareness and understanding of illness.”[3]
- “A person’s capacity to understand the nature, significance and severity of his or her own illness.”[4]

Grades of insight ranging from complete denial to true emotional insight have been suggested.[5] These grades include

- Complete denial of illness
- Slight awareness of being sick and needing help but denying it at the same time.
- Awareness of being sick but blaming it on others, external factors, or medical or unknown organic factors.
- Intellectual insight: Admission of illness and recognition that symptoms or failures in social adjustment are due to irrational feelings or disturbances, without applying that knowledge to future experiences.
- True emotional insight: Emotional awareness of the motives and feelings within and of the underlying meaning of symptoms, whether the awareness leads to changes in personality, and future behavior; openness to new ideas and concepts about self and important people in the person’s life.

Insight has implications for and impact on the patient’s life, functioning, and on treatment compliance.

### Traditional definition and implication

Traditionally insight has been defined as “the correct attitude to morbid change in oneself and the realization that the illness is mental”.[6] Jaspers had also discussed insight in similar terms.[7] The traditional point of view supports the contention that insight is absent in psychosis and that it is an all or none phenomenon. For example, 97% of patients examined in the International Pilot Study of Schizophrenia did not have insight.[1]

The acknowledgement of a mental illness by the patient was cardinal to diagnosis of the presence of insight. Insight is briefly assessed by instruments such as the positive and negative syndrome scale.[8] The absence of insight was considered an important part of all psychoses including schizophrenia. The categorical assessment of the phenomena also resulted in many people with schizophrenia not meeting the required standard.

### Current views on insight

The traditional uni-dimensional view of insight has been replaced with recent multi-dimensional perspectives.[9] Three dimensions of insight have been recognized: (i) awareness of mental illness, (ii) ability to re-label psychotic experience as abnormal, and (iii) seeking medical treatment. Instruments have been devised to assess and quantify insight [e.g. the insight and treatment attitudes questionnaire,[10] the schedule for assessment of insight,[11] and scale to assess unawareness of mental disorders.[12] These instruments have been employed to assess insight and its association with psychopathology, social functioning, and prognosis. However, these studies have been mainly done in western populations.

The recent multi-dimensional perspectives of insight have also been criticized.[13–15] The major criticism is that these perspectives employ western concepts of disease. All mental illnesses are considered medical diseases and a failure to subscribe to such a point of view often result in a diagnosis of the absence of insight. Alternative local and culture explanations for mental illness are discounted. Non-western beliefs are excluded from being standards in parts of the non-western world. Many workers have argued that the current perspectives represent the arrogance of bio-medical views. Others have suggested that the cost of labeling and stigma related to mental disorder label are high and unacceptable.

### Work from India

The *Indian Journal of Psychiatry* was searched for the term “insight” while PubMed was explored using the terms “insight”, “schizophrenia”/ “psychosis”, and “India”. The relevant articles retrieved. This paper presents an overview of some of the work from India and argues for an alternative conceptualization of insight in psychosis.[16–37]

### Studies of illness perspectives and on insight

Illness perspectives (also called explanatory models) among patients suffering from a variety of conditions have been the focus of recent studies. Explanatory models (EMs) denote the “notions about an episode of sickness and its treatment that are employed by all those engaged in the clinical process.”[38] *Emic* models elicit patient perspectives by the way patients conceptualizes their sickness episode including beliefs and behaviors concerning etiology, course, timing of symptoms, meaning of sickness, diagnosis, methods of treatment, roles, and expectation of sick individuals.[38,39] EMs influence many aspects of the human behavior like help seeking, treatment compliance, patient satisfaction, and coping.

Explanatory models have been examined in the following populations: (i) patients with tuberculosis,[16] (ii) patients with unexplained medical symptoms,[17,18] (iii) patients attending traditional healers,[19] (iv) patients with psychosis, and[20] (v) explanatory models of psychosisamong community health workers.[21,22] The explanatory models of illness and issues related to insight have been assessed in patients with schizophrenia[23–31] and with bipolar disorders[25,32] and their relatives. The findings of these studies suggest the following broad conclusions. (i) Patients, relatives, and health workers often provide non-medical explanations for the cause of illness (e.g. karma, evil spirits, black magic, sin, punishment by god, etc). (ii) Many patients and relatives hold multiple beliefs including medical, non-medical, supernatural, religious, and black magic. These beliefs are simultaneously held and are often contradictory. (iii) Many patients (and their relatives) simultaneously seek bio-medical and non-biomedical interventions. (iv) Insight scores and the severity of psychopathology are not significantly or only partially correlated.[33–36] (However, correlation between changes in psychopathology and insight are reported to be significantly related to outcome at 1-year follow-up[36]) (v) Some studies have not shown a correlation between insight and medication compliance.[37]

These findings argue that the current multidimensional models to assess insight are not culturally sensitive. It suggests that it is difficult to have universal measures for insight. It argues that there is a need to compare individual explanations with sub-cultural perceptions.

### The analogy with assessment of delusions

Similar comparisons with local beliefs are standard in the current schemes, which assess other clinical phenomena.[30] For example, delusions are considered false based on rational thought or considered abnormal if they are not shared by the culture or a religious subgroup.[40] Consequently, an understanding of the local culture is mandatory in delineating clinical phenomena like delusions. The need to use local standards rather than universal yardsticks to assess delusions in people with psychosis is an accepted norm in clinical psychiatry. A similar situation exists as far as the assessment of insight is concerned. Norms for insight based entirely on western notions of health and illness are invalid in non-western societies. Consequently, local standards need to be employed in the assessment of insight in people with psychosis.

### Proposed changes to the standard

The following changes in the dimensions of insight have been proposed [Table 1].[30] If a person acknowledges some kind of non-visible change in his or her body or mind that affects the ability to function socially, and if he or she feels the need for restitution, then, irrespective of the attribution and the pathways of care that the person seeks, we could call this the presence of “insight”. This would suggest the need to use local and cultural standards rather than universal yardsticks to assess insight in people with psychosis. The awareness of changes in body or mind has to have a non-delusional explanation. Delusional explanations would have to be excluded as they are part of the disease and would differ from local cultural explanations. A detailed comparison with explanations of similar illness with those of family and local culture is mandatory to arrive at such a conclusion.

**Table 1:** Proposed changes to dimensions of insight[30]

Current dimensions of insight	Proposed dimensions of insight
Awareness of mental illness	Awareness of non-visible change in body or mind and its relation to their illness
Re-label experience	Re-label experience
Seek medical treatment	Need restitution and seeks any forms of available treatment

Diagnosing the non-delusional nature of the explanation requires an understanding of the local culture.[30] For example, black magic is a commonly held belief among the general population in India. The belief in black magic held by the vast majority of people in India is general in nature and is not directed specifically at any person or groups of people. The remedies to overcome such influence are also general in the form of rituals and charms to ward of such effects. On the other hand, delusions of control and persecution through black magic that are specifically directed at individuals or a group of people are not shared by the cultural subgroup and their explanations suggest abnormal thought processes. Consequently, recognizing delusions of control and persecution based on black magic in people with psychosis would require an understanding of the local culture.

The belief should be assessed comprehensively. For example, what is the strength of the conviction? What is the extent of the belief? Does the explanation reveal abnormal thought processes? Does the person act on the belief? What is the impact of the belief on the person’s life? Has there been a qualitative and quantitative change in the belief over time? The answers to these questions would necessarily have to be compared with responses of people from the patient’s culture before it is considered as a phenomenon of psychosis. On the other hand, non-delusional explanations, which correspond with those of the subculture, would suggest the presence of insight.

The proposed concept of insight [Table 2][30] can be said to have two aspects for its different components: awareness and attribution or action. People with psychosis can be aware of changes in body or mind and can re-label their psychotic experiences as different or abnormal. Such an acknowledgement would suggest insight. Attributing such changes to explanations commonly present in local culture would also imply the presence of insight. However, these explanations would necessarily have to be non-delusional. They will have to be compared with that held by the subculture in order to rule out the possibility of it being a delusional component of the illness. In addition, being aware of the locally available solutions to such problems would add to the evidence that the person is aware of the local reality. Acting on such attributions and seeking locally available help definitely suggest insight. The awareness, attribution, and action related to the different components of insight suggest that it is a complex phenomenon. People with psychotic illness can have different grades of insight.

**Table 2:** Proposed scheme to assess insight[30]

Component	Awareness	Attribution/action
Psychotic experiences	Awareness of difference/re-labeling of experience	Attribution corresponds with local cultural explanations for illness
Change in body or mind	Awareness of change	Attribution corresponds with local cultural explanations for illness
Seeks available treatment	Acknowledgement of need for restitution	Seeks available intervention

### Explanatory models in western countries

There is some evidence to suggest that patients in other cultures also simultaneously hold multiple and possibly contradictory beliefs. Lloyd *et al*.[41] studied explanatory models of common mental disorders among different ethnic groups in Britain (White, Afro-Caribbean and Asian). The results suggest that multiple and contradictory explanations may be held simultaneously by different ethnic groups. Similarly, a study of explanatory models in people with schizophrenia living in the London also reported diverse and multiple models of illness.[42] Differences between models held by different ethnic groups were also documented. These differences argue that the immediate subculture seems to play a big role in determining explanatory models of illness. While people with psychosis may be aware of and re-label their psychotic experiences, their attributions and actions may be influenced by their local culture and background. The results of these studies suggest the need for comparing the beliefs held by patients with that of the subculture in order to ascertain the similarities in the explanations of the illness.

### Universal conventions to assess insight

Explanatory models of illness are often multiple and it is difficult to reduce them to simple categories. In addition, they are fluid and dynamic and may change over time. Most cultures offer more than one explanation for psychosis and also provide diverse sources of help.[30,43–45] While antipsychotic medication has a powerful impact on the outcome of schizophrenia, other forms of psychological inputs (e.g. counseling, religion, help with social and occupational problems, social support, psychological help using a variety of 𠆉native treatments’) are also useful. While such non-psychotropic medication inputs may have a less powerful impact on course and outcome, those seeking such interventions may be aware of their illness, realize the need for restitution, and seek locally available therapy suggesting that they do possess insight.

Just as local standards are employed in the assessment of delusions around the world, the same “rules” should be applied to assess insight in people with psychosis across cultures. People with psychosis who are able to re-label their psychotic experience, offer non-delusional explanations for changes in themselves, which correspond to beliefs about illness held by the subculture, admit to the need for restitution, and seek locally available help, can be said to possess insight. The complex nature of the assessment implies that there are grades of insight. However, the same conventions can be employed across cultures to assess it.

### Implications of findings

The implications of these findings include the following:

1. The need to accept multiple approaches to restoring health;
2. The need to understand the patient’s perspective;
3. The need to explore different dimensions of patient experience;
4. The necessity to integrate the apparent contradictions between the biomedical and non-medical beliefs;
5. The requirement to emphasize role of medication compliance;
6. To encourage use of diverse strategies to restore and improve psychological health and functioning; and
7. To reduce stigma related to mental illness (so that people can identify with the medical model).

Educational programs for patients, their families, and the community should elicit explanatory models commonly prevalent in local communities. Such programs should put forward the biomedical perspective without dismissing or directly challenging local beliefs. The education package should cover a variety of topics including symptoms, local beliefs about causation, biomedical models, psychosocial influences, prevalence, diagnosis, treatments including medication and compliance, side effects of medication, role of hospitalization, and coping strategies for families. While the patients and their families are encouraged to comply with medication schedules, they can also be encouraged to seek non-medical interventions as these have a powerful impact on their psychological health.

## CONCLUSION

Insight in psychosis is a complex issue. The results of these studies suggest that it is difficult to have a universal measure for insight. The findings argue for the fact that the assessment of insight should be against the local cultural standards rather than universal yardsticks.
